# Phage Therapy in the Year 2035

**DOI:** 10.3389/fmicb.2020.01171

**Published:** 2020-06-03

**Authors:** Jean-Paul Pirnay

**Affiliations:** Laboratory for Molecular and Cellular Technology, Queen Astrid Military Hospital, Brussels, Belgium

**Keywords:** infectious diseases, antibiotic resistance, antimicrobial resistance, phage therapy, synthetic biology, artificial intelligence, machine learning, distributed ledger technology

## Abstract

The emergence of multidrug resistant bacteria in both community- and hospital-acquired infections is recognized as a major public health threat. Phage therapy is increasingly mediatized and researched as an additional tool for combatting antibiotic resistant infections. However, phages exhibit a number of properties that differ from antibiotics and hamper their development as pharmaceutical products and their application in therapy. This paper advocates a paradigm shift in the development and application of infectious disease therapeutics to cater for personalized phage therapy, which could be realized by the year 2035. More specifically, it presents a sustainable and ethical supply chain of instant synthetic phages, based on a community effort, supported and steered by public health organizations, and managed by a platform combining Artificial Intelligence (AI) and Distributed Ledger (DL) Technology.

## Preface

This paper offers a personal vision of what might be needed for phage therapy to finally break through as a mainstream antibacterial tool. It is influenced by historical and recent failures and uncertainties in the phage therapy field and aims at finding solutions based on future and emerging technologies that are supposed to model the science and society of tomorrow.

## Phage Therapy

Bacteriophages (phages) are the viruses of bacteria. Since time immemorial they have controlled the growth and spread of their bacterial hosts. Bacterial viruses are the most ubiquitous lifelike entities in our biosphere. There are an estimated 10 million-fold more viruses in the oceans than there are stars in the universe and if all the phages on Earth were stacked on top of another, this tower would stretch further than the nearest 60 galaxies (Suttle, 2013). They can easily be found wherever bacteria thrive: in sewers, rivers, or patients’ urine and stool. Phages of human bacterial pathogens are most often composed of an icosahedral head, a sphere with 20 flat faces made of proteins and containing a nucleic acid genome, to which a protein tail is attached. When a strictly lytic phage adheres with its tail fibers and spikes to the surface of its target bacterium, the syringe-like tail sheath contracts and the tail core is driven through the bacterial cell wall, injecting the phage genome into the periplasm of the bacterial cell. Immediately, the bacterial DNA and protein synthesis machinery is hijacked to build copies of the phage. Some phages cut the bacterial DNA into pieces. After a latent period of minutes to hours, the newly formed phages burst out of their bacterial hosts, which are killed in the process. The phage progeny, which can run into the hundreds per bacterium, then go off to find new host bacteria to infect. As such, phages can be considered as self-replicating antimicrobials. Importantly, phages have evolved to only infect certain target bacteria and are harmless to mammalian cells.

Early evidence of viral-like agents with antibacterial activity was reported by the English bacteriologist Frederick Twort, and by the French-Canadian microbiologist Felix d’Hérelle in 1915 and 1917, respectively (Sulakvelidze et al., 2001). In 1919, d’Hérelle exploited for the first time the therapeutic potential of phages when he used them to cure a boy suffering from dysentery in Paris. Phage therapy was immediately recognized as a therapeutic approach to treat bacterial infections and commercialization of phage therapy preparations was undertaken by several companies, such as L’Oréal in Europe and Eli Lilly Company in the United States (Sulakvelidze et al., 2001). In 1923, the Georgian microbiologist Giorgi Eliava founded the Eliava Institute in Tbilisi, Georgia, devoted to phage therapy research. It was the start of extensive phage therapy research and development in the former Soviet-Union. However, early uses of phage therapy were often unreliable and research into antibiotics had also been ongoing. The successful use of penicillin during the Second World War and its subsequent worldwide marketing led Western scientists to lose interest in phage therapy. Soviet researchers, in contrast, continued to develop phage therapy and to publish their results, but due to the Iron Curtain their knowledge and experience did not spread across the world (Sulakvelidze et al., 2001). At the dawn of the third millennium, the increasing health burden of infections with antibiotic resistant bacteria (Cassini et al., 2019) incited a renewed worldwide interest in phage therapy as a viable additional tool to the clinical management of bacterial infections (Thiel, 2004). All over the world, phage therapy centers are being set up, following in the footsteps of the Eliava Institute and the phage therapy unit at the Hirszfeld Institute in Wrocław, Poland (Miêdzybrodzki et al., 2012).

## The Year 2035

Fast forward to future Earth of 2035, a gloomy world characterized by human overpopulation, major ecosystem disruptions, global warming, and xenophobia.

While soaking in his bath, Dr. John Iverian, a retired microbiologist, suddenly felt an extremely painful sting in the back of his neck, followed by a sound like a small plane’s propeller. He screened the environment and in the corner of his eye he saw a weird large insect with long creepy legs and antennas sitting on the wall next to his designer bathtub. Osuri, the home management system of Iverian’s loft in the center of Antwerp, identified the insect as the brown marmorated stink bug *Halyomorpha halys*, which had spread across the world. Osuri’s report, projected on one of the bathroom’s video screens, mentioned that people, who were bitten, initially experienced a small red sore in the bite area of their skin. When left unattended, the bite wound would swell and produce puss. Tired and muzzy, non-chalant Iverian stepped out of his bath and went to bed. He had decided not to perform the elaborate wound treatment procedure, which had strongly been advised by Osuri. Early next morning, however, the bite had turned into a necrotic wound showing clear signs of infection.

Anxiously, Iverian activated his Phage-BEAM device. BEAM stood for “Bedside Energized Anti-Microbial.” The device had the size and shape of a shoebox, but with a more elegant and polished look. The name of the device and its manufacturer were designed in colorful letters on the side of the seamless white enameled box. Iverian removed a swab from its sterile packaging and gently passed it over the entire area of the wound, making sure that the wound exudate thoroughly wetted the cotton wool tip of the swab. When the swab approached the “insert sample” area of the box, a tiny door opened as if magically, freeing a 10-inch high hologram of a lab technician, named Marcia. She showed Iverian where to dock the sample. Marcia was developed to guide the clients through the test procedure. “For best results, please insert a new phage bio-ink cartridge, Dr. Iverian,” Marcia said. Just as it used to be for yesteryear’s 2D printers, the cost of the bio-ink cartridges was almost as high as the cost of the Phage-BEAM device itself. According to “Business Insider,” phage bio-ink was the second most expensive liquid on Earth, behind Chanel No. 8. Luckily, as one of the inventors, Iverian had obtained the right to always have the most recent version of this device at his disposition, including a continuous supply of reagents, for free.

Iverian knew perfectly how the device worked, so he did not need Marcia’s help. First, DNA was extracted from the swab tip and the metagenome—all the genetic material present in the sample, including the infecting bacteria—was determined. Next, these genetic data were sent to a secured “Phage XChange” server where a complex AI-driven algorithm predicted the genome sequence of the phage that was most likely to lyze the infecting bacteria identified in the metagenome and was supposed to elicit the weakest immune reaction in the patient. The phage genome data were sent to the Phage-BEAM device, which first synthesized the phage genome and then the phage, using a proprietary bacterium-free phage production system.

Within 1 h after sampling, the device would produce a ready-to-use therapeutic phage product. Results of the step-by-step procedure would be transmitted to the enormous home video screen in Iverian’s living room. Sitting in his LC2 armchair, listening to Mozart’s Great Mass in C minor, Iverian anxiously waited for the results to come in. He had a bad feeling about this. The result sent shivers down Iverian’s spine. Bacterial pathogen identified: *Streptococcus pyogenes* strain FE-2033! Osuri immediately activated the infection alert protocol, sending a message to the World Center for Disease Control and projected worrisome background information on the lethal flesh eating bacterial strain, which was considered an imminent threat to public health since 2033. For a moment, Iverian considered excising the infected wound and some surrounding healthy tissue with a kitchen knife, but he calmed down and decided to wait and apply the imminent Phage-BEAM product. An hour later, the Phage-BEAM device had produced synthetic phages. These phages were then mixed with the isolated bacteria, in a validation module, to test their *in vitro* efficacy. Fifteen minutes later, the green light was given for Iverian to commence treatment. Iverian applied the phages in a slow release hydrogel-based wound dressing, which had first been mixed with the concentrated phage suspension produced by the Phage-BEAM device, and also contained synergistic antibiotics. The hydrogel temporarily relieved the pain, which further calmed him. Iverian repeated application of the phage and antibiotic-loaded hydrogel once a day. Wound infection improved within 24 h and after 7 days the wound was almost completely healed. Iverian’s potentially life-threatening infection was successfully treated, timely and without leaving his home. But, for many previous decades, it had not been certain that phage therapy would break through to become a broadly applied and clinically useful antibacterial tool. The medical world had taken a while to realize that phage therapy did not need to be identical to antibiotic therapy, and this mainly because of the peculiarities of the active agents, the phages.

## Some Relevant Peculiarities of Phages

Phages exhibit a number of properties that differ from antibiotics and hampered their development as pharmaceutical products and application in therapy. First, they tend to be specific about which bacteria they infect. They will at best target a considerable part of one single bacterial species, but at worst they will infect only a small number of strains within one species. Therapeutic phages can thus be selected to mainly kill one bacterial species, or a clinically relevant subgroup thereof, and spare the patient’s beneficial bacteria (e.g., the gut, skin, or oral commensal flora). Most routinely employed antibiotics, in contrast, have a broad spectrum of activity, which can cause “collateral damage” to the patient’s commensal microbiomes, which in turn can result in adverse effects such as the selection of antibiotic resistant bacterial species (e.g., *Clostridium difficile*) or antibiotic-associated diarrhea (Jernberg et al., 2010). The drawback of phage specificity is that the infecting bacteria need to be identified before starting phage therapy. In empirical antibiotic therapy, in contrast, broad-spectrum antibiotic cocktails that affect a multitude of Gram-positive and Gram-negative bacteria, and diverse fungi are typically used. When more information is known (e.g., from bacterial culture), treatment may consist of narrow-spectrum antibiotics, which more specifically target the bacteria or fungi identified to be causing disease.

Second, bacteria and phages are involved in a host–parasite relationship. Strictly lytic phages are ubiquitous in the environment and require the death of their bacterial host to complete their life cycle. Without hosts, phages cannot exist. Phages impose selection for resistant hosts, which in turn impose selection for effective phages. This results in what is called “antagonistic coevolution,” an arms race between bacteria and phages, characterized by reciprocal evolution of bacterial resistance and phage infectivity (Buckling and Rainey, 2002). Just as with most antimicrobials, bacteria will thus also become resistant to phages (Luria and Delbrück, 1943; Schooley et al., 2017), but, in contrast to static antibiotics, phages have the capacity to overcome bacterial resistance (Buckling and Rainey, 2002). There are nevertheless indications that bacteria and phages will not indefinitely increase their respective resistance and infectivity (Fortuna et al., 2019).

## Phage Therapy Approaches

At the time of the phage therapy revival in the early 2000s, two distinct phage therapy approaches had been developed (Pirnay et al., 2011). In what could be called the *one-size-fits-all* approach, defined broad-spectrum phage cocktails, which were supposed to target the majority of bacteria suspected to cause certain infectious diseases, were applied. These predefined broad-spectrum phage cocktails were developed, produced, and tested within the current pharmaco-economic models, which had been designed to cater for “static” drugs such as antibiotics. However, truly broad-spectrum phage cocktails, active against most Gram-positive and/or Gram-negative bacteria commonly encountered in infectious diseases needed to contain large amounts of phages and turned out to be very difficult to develop. It was feasible to develop narrower spectrum phage cocktails, active against one or a few bacterial species, to be used in certain indications and minding that the infecting bacterial species were known in advance. For some bacterial species, such as *Staphylococcus aureus*, phages showing an exceptionally broad host range had been isolated and characterized (Vandersteegen et al., 2011). In PhagoBurn, a randomized controlled trial, success in achieving sustained reduction in *Pseudomonas aeruginosa* burdens in burn wounds was linked to initial susceptibility to the phage cocktail (Jault et al., 2019). However, one-third of the included patients were shown to harbor pre-existing *P. aeruginosa* strains resistant to the phage cocktail, which consisted of no less than 12 lytic *P. aeruginosa* phages. In addition, phage cocktails that were initially designed to be effective needed to be regularly updated (e.g., supplemented with new phages) in response to the emergence of phage resistance or the involvement of newly circulating clinically relevant strains. Finally, it was not known if confronting bacteria with high concentrations of fixed phage cocktails would cause the emergence, spread and persistence of bacterial phage resistance in hospitals and in the environment, similar to what had happened upon the massive use of antibiotics.

**FIGURE 1:**
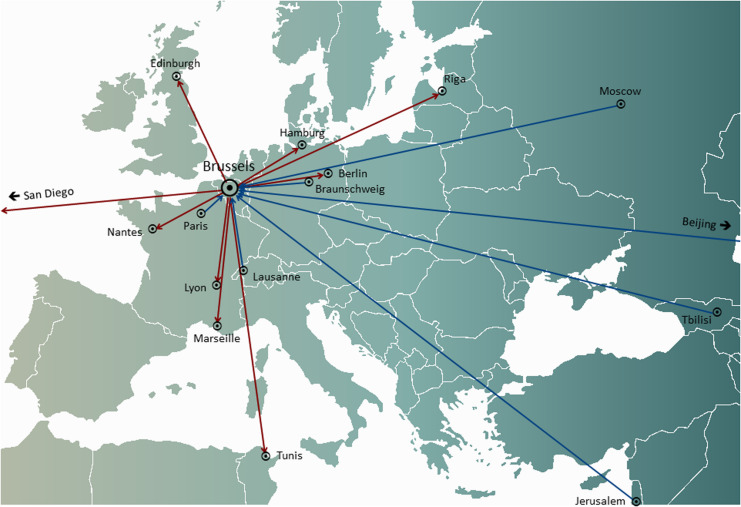
International transfers of phages from (red arrows) and to (blue arrows) the Queen Astrid Military Hospital (QAMH) in Brussels in view of clinical applications over the period 2015–2020. On the national level, phages were dispatched from the QAMH to five university hospitals (not shown). In addition, the selection of matching phages often encompassed the transfer of the patients’ bacterial isolates, and five international patients (two from France, two from the Netherlands, and one from Tunisia) were transferred to Brussels for phage therapy.

In *personalized* phage therapy concepts, one or more phages were selected from a phage bank, or from the environment, and possibly adapted (*in vitro* selection of phage mutants exhibiting increased infectivity) to more efficiently infect the bacteria isolated from the patient’s infection site (Friman et al., 2016). Some phage therapy centers set up and maintained large therapeutic phage banks, which were regularly updated with new phages, widening and adapting the host range of the bank to the ever-changing bacterial populations. Personalized phage therapy approaches were potentially more sustainable, as only the infecting bacterium is targeted, resulting in less selection pressure toward bacterial phage resistance. However, they were also more elaborate and logistically complex than one-size-fits-all approaches, with bacterial strains and matching phages being sent all over the world (Figure 1). Moreover, precision medicine concepts were, in general, not compatible with most medicinal product (drugs in the United States) development and licensing pathways, which required several years and millions of euros (dollars) to complete, and this for every phage in the bank (Verbeken et al., 2012).

## Enter Synthetic Biology

With the onset of the third millennium, synthetic biology approaches had been increasingly developed to reduce the specificity of phages and the emergence of bacterial phage resistance (e.g., structure-guided design) (Pires et al., 2016; Dunne et al., 2019). For instance, yeast-based platforms for phage tail fiber protein switching were elaborated to engineer hybrid phages with more predictable and extended host range (Ando et al., 2015; Yosef et al., 2017) and genetic engineering strategies (e.g., CRISPR-Cas editing tools) were developed to address other aspects such as negative patient-phage interactions (e.g., anti-phage immune response) (Brown et al., 2017), the potential emergence and spread of bacterial phage resistance mechanisms, and the release of harmful bacterial contents such as endotoxins (Hwang et al., 2018). Synthetic phage genomes needed to be rebooted to produce phage offspring (Barbu et al., 2016; Pires et al., 2016), through transformation of *Escherichia coli* or *Listeria monocytogenes* L-forms (Kilcher et al., 2018), or using cell-free transcription-translation (TXTL) systems (Rustad et al., 2018). Western regulatory frameworks had gradually started to cater for precision and personalized phage therapy approaches using naturally occurring phages (Pirnay et al., 2018), engineered phages (Dedrick et al., 2019), and synthetic phages.

The development of *ad hoc* and on-site therapeutic phage production devices, such as Phage-BEAM, did not run smoothly, Iverian recalled. To start with, it required artificial intelligence (AI)-based *in silico* phage matching and design. Deep learning (Martorell-Marugán et al., 2019), a subset of Machine Learning, was chosen to search for links between bacterial genomes and infecting phage genomes, because it was easier to scale to bigger number of samples. For instance, deep learning methods did not require so-called feature extraction, which would require gene/protein level annotation of phage and bacterial genomes and would limit predictions to certain known relationships between bacterial and phage features, such as phage tail fiber structures binding to specific bacterial cell wall receptors. As a down side, it needed to be powered by a continuous supply of massive amounts of data, linking lytic phage genomes to host bacterial genomes, and that’s where the shoe pinched. Whole genome sequencing had slowly percolated into the practice of clinical microbiology (American Academy of Microbiology, 2016), but research institutes and pharmaceutical companies were not keen to submit their data to a single centralized database, and no investors were found willing and able to acquire the available data and/or to produce sufficient amounts of new data. A second obstacle that had to be overcome was the unavailability of quick, reliable, and affordable synthesis of large DNA molecules. Initial DNA synthesis techniques were based on organic chemistry and produced relatively small DNA molecules. The *de novo* synthesis of phage genomes required assembling several genome fragments (Barbu et al., 2016; Pires et al., 2016; Lemire et al., 2018) in the yeast *Saccharomyces cerevisiae*, using yeast artificial chromosomes (Ando et al., 2015), or chemical assembly (Gibson et al., 2009). The development of a new technique to synthesize DNA, based on DNA-synthesizing enzymes found in cells of the immune system (Palluk et al., 2018), facilitated phage genome synthesis. Finally, some hurdles had to be overcome to develop generic cell-free phage production systems able to produce phages in high titers and exhibiting the same levels of bacterial infectivity as their natural analogs.

## The Breakthrough

**FIGURE 2:**
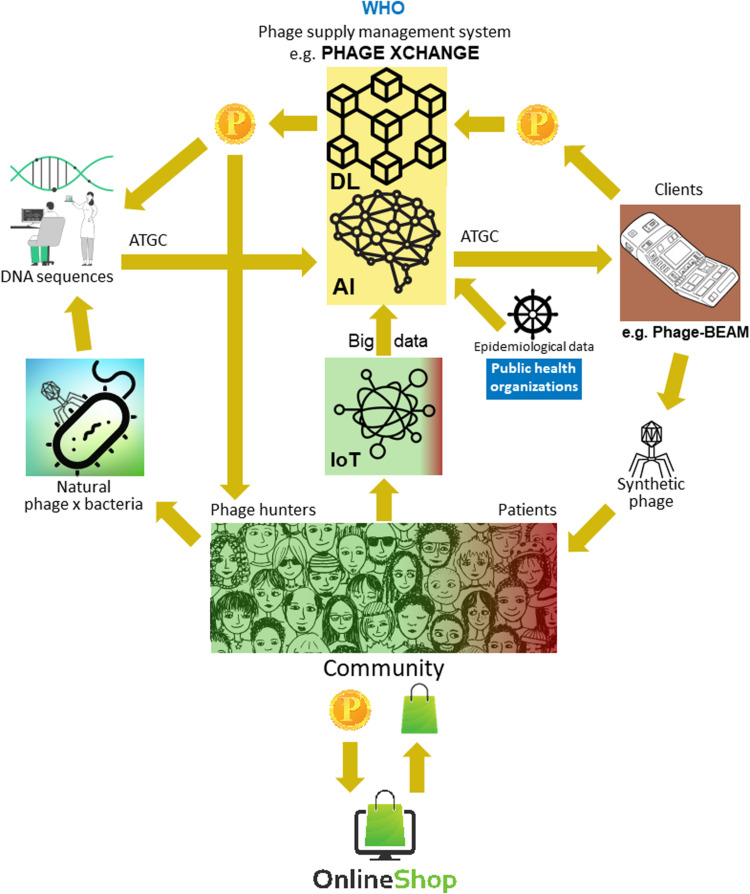
Vision of how the phage supply chain might be organized in 2035. AI, artificial intelligence; ATGC, DNA sequence; BEAM, bedside energized anti-microbial; DL, distributed ledger; IoT, Internet of Things; P, PhageCoin; WHO, World Health Organization.

The major problem was that it turned out to be very difficult to collect the massive amounts of linked phage and bacterial genome sequences necessary for the deep learning AI algorithms to predict and/or design phage sequences with a therapeutically acceptable level of accuracy. Iverian remembered that the real breakthrough came when the not-for-profit organization “Phage XChange” launched its global phage governance platform of the same name to create an efficient, standardized, sustainable, and ethical phage supply chain (Figure 2). Phage XChange mainly consisted of an AI module and a Distributed Ledger (DL) (Thiebes et al., 2020). The platform’s AI module analyzed linked phage and bacterial genomes to predict and design potent phages for clients. It also predicted which bacterial pathogens needed the most urgent attention, based on the Internet of Things (IoT) and Big Data and information provided by international public health organizations, such as the World Health Organization (WHO) and national Centers for Disease Control. These data steered the system toward the isolation and characterization of the most urgently needed phages.

The platform’s DL module ensured a sufficient, qualitative, and recorded input of linked phage/bacterial genome sequences to the AI module and ditto supply of phage sequences to clients, in compliance with the provisions of the Nagoya protocol (Expert round table on acceptance and re-implementation of bacteriophage therapy et al., 2018). The DL immutably recorded all stakeholder (e.g., suppliers of data, sequencing services, and clients), transaction, and contract details. It also recorded the exact quality, specifications, and weight of the supplied material. An algorithm determined the non-redundancy and estimated the weight (e.g., the virulence and host range of the phages) and desirability of the submitted material. Phages targeting emerging bacterial pathogens were of course most wanted. Most patent issues were obviated. The DL acted as a payment ledger to assure that all parties were paid timely and fairly. A number of PhageCoins (the platform’s crypto currency) were attributed to the suppliers in relation to the quality, weight, and desirability of the supplied material. Clients extracting prediction results (phage sequences) through the DL paid an amount of PhageCoins, proportionate to the estimated value of the phages. These PhageCoins were used to maintain the DL, to assure a sufficient and continuous inflow of material, and to expand phage virulence and host range data (matching phages to bacteria). An additional injection of funds and incentive to supply material was found in producers and suppliers of all kinds of goods. With the instantly earned PhageCoins, phage suppliers could buy online all kinds of products at strongly reduced prices, from laboratory- and school equipment to sports items. These goods were provided through corporate sponsorship. Several established companies sponsored PhageXchange in exchange of tax reductions, publicity, and the image of a socially responsible brand. The weight of the supplied material, and thus also its value, were initially undervalued, but were re-evaluated at regular intervals (iteration) and suppliers were attributed more PhageCoins when warranted. Even though useful from the moment it was introduced, the platform only became really successful when it was put under the protection of the WHO, in analogy to the worldwide system of traceability, transparency, vigilance, and surveillance of Medicinal Products of Human Origin (Warwick et al., 2013). A formal agreement between Phage XChange and the WHO increased international confidence in the long-term sustainability of the platform and protection from unethical commercial exploitation. The search for potent therapeutic phages soon became a community effort aimed at solving the antibiotic resistance crisis, with independent “phage hunters,” schools, scout groups, villages on the banks of the Amazon River, etc., isolating and submitting phages to Phage XChange, in exchange of PhageCoins. At the margins of this, various companies and institutions developed peripheral equipment and services, such as phage isolation kits and sequencing and phage synthesizing platforms (e.g., the Phage-BEAM device). In anticipation of these devices, intermediary solutions were offered, whereby the phages themselves were obtained through the DL.

## Epilog

This view on the future of phage therapy provides an optimistic ending to the antibiotic resistance crisis. The *ad hoc* and on site production of synthetic phages, linked to a global, community-based, phage management system, turned out to be a welcome and affordable (for social security systems) extra weapon in the fight against antibiotic resistant bacterial infections. However, it was not a magic bullet; it was a synergistic supplement to established antimicrobials. The instant and cell-free production of synthetic phages, whether designed or not, had considerable advantages over classically produced (in bacterial hosts) natural phages:

(i) There was no need to maintain physical therapeutic phage banks and to dispatch the patient’s bacterial isolates and the matching therapeutic phages all over the world.
(ii) Synthetic phages against bacteria causing eminent public health threats, such as the 2011 *E. coli* O104:H4 outbreak in Germany (Merabishvili et al., 2012), or bacteria (suspected to be) used for bioterrorism (Joñczyk-Matysiak et al., 2014) could be timely produced on site.
(iii) Phages against bacteria causing potentially lethal diseases, for which no non-lethal production host strains were available and whose propagation used to require biosafety level-3 (bsl-3) bio-containment precautions, could be synthesized in bsl-1 conditions.
(iv) When no phages could be isolated from sampling sites, for instance, because the bacterial host strains used in the isolation techniques were not susceptible to the desired phages, (predicted) phage genomic sequences, extracted from metagenomic data (Reyes et al., 2010; Amgarten et al., 2018), could be used to produce synthetic phages.
(v) Synthetic phage preparations contained no (or smaller amounts of) molecules that could have a negative impact on patients (e.g., endotoxins).
(vi) Devices were adapted to produce synthetic phages during extended space travel and space colonization (Taylor and Sommer, 2005).

There is little chance that these predictions will come true. It is probably too shortsighted to think that a community-based effort, supported by public health organizations and managed by a global, sustainable and ethical platform, could be at the heart of a solution to the current worldwide antibiotic resistance crisis. Some parts of the proposed system, such as cell free production of synthetic phages using a bedside device, have a reasonable chance of being realized, while other elements, such as corporate sponsorship, will likely remain limited to the realm of science fiction. You may say that I’m a dreamer, so feel free to wake me up in 2035 to confront me with reality!

## Author Contributions

J-PP conceived the vision and drafted the manuscript.

## Conflict of Interest

The author declares that the research was conducted in the absence of any commercial or financial relationships that could be construed as a potential conflict of interest. The handling editor declared past co-authorship with the author.
